# Single Position ECG Detection System Based on Charge Induction

**DOI:** 10.3390/s23104771

**Published:** 2023-05-15

**Authors:** Yi Yang, Kun Xu, Yu Li, Yahui Zhang, Limin Zhang

**Affiliations:** School of Electronic Science and Engineering, Nanjing University, Nanjing 210023, China; mf20230136@smail.nju.edu.cn (Y.Y.); kxu@smail.nju.edu.cn (K.X.); mf21230061@smail.nju.edu.cn (Y.L.); mf21230166@smail.nju.edu.cn (Y.Z.)

**Keywords:** single position, one electrode, charge induction, ECG detection

## Abstract

With the growing incidence of cardiovascular disease (CVD) in recent decades, the demand for out-of-hospital real-time ECG monitoring is increasing day by day, which promotes the research and development of portable ECG monitoring equipment. At present, two main categories of ECG monitoring devices are “limb lead ECG recording devices” and “chest lead ECG recording devices”, which both require at least two electrodes. The former needs to complete the detection by means of a two-hand lap joint. This will seriously affect the normal activities of users. The electrodes used by the latter also need to be kept at a certain distance, usually more than 10 cm, to ensure the accuracy of the detection results. Decreasing the electrode spacing of the existing ECG detection equipment or reducing the area required for detection will be more conducive to improving the integration of the out-of-hospital portable ECG technologies. Therefore, a single-position ECG system based on charge induction is proposed to realize ECG detection on the surface of the human body with only one electrode with a diameter of less than 2 cm. Firstly, the ECG waveform detected in a single location is simulated by analyzing the electrophysiological activities of the human heart on the human body surface with COMSOL Multiphysics 5.4 software. Then, the hardware circuit design of the system and the host computer are developed and the test is performed. Finally, experiments for static and dynamic ECG monitoring are carried out and the heart rate correlation coefficients are 0.9698 and 0.9802, respectively, which proves the reliability and data accuracy of the system.

## 1. Introduction

Health awareness has grown exponentially over the past few decades, while medical advances have revolutionized the treatment of a variety of key diseases. However, most of the equipment used to detect patients in hospitals is not portable and needs to be operated by medically trained experts, so patients cannot observe their disease in real time after leaving the hospital. In order to continuously track patients’ physical activities and physiological parameters in the out-of-hospital environment, a wearable health monitoring system has been developed [1]. Wearable systems are mainly used for the monitoring or investigation of cardiovascular diseases, such as cardiac arrest, arrhythmia, congestive heart failure, coronary artery disease, etc. [2].

As one of the most widely used vital signs, the electrocardiogram (ECG) provides useful diagnostic information of the cardiovascular system. It can be used as a strong indicator of certain physiological and pathological conditions in humans. At present, the ECG detection equipment outside the hospital can be divided into invasive and non-invasive. For example, the implantable loop recorder is an alternative system with an electrode distance of 4 cm [3] which can obtain an accurate ECG signal, although it is more invasive and expensive. The non-invasive ECG detection equipment mainly falls into two categories, which both require at least two electrodes. One is called “limb lead ECG recording devices”, which uses the fingers, wrists, or palms of both hands to obtain ECG signals. The other can be called “chest lead ECG recording devices”. These devices are usually embedded in wearable clothing or a band and use electrodes attached to the chest to capture two or more electrocardiogram leads.

Handheld ECG devices and smartwatches are two of the most common applications of “limb lead ECG recording devices”. As one of the most representative handheld devices, AliceCor Kardia (ACK), Mountain View, CA, USA, is a smart phone ECG device that allows users to perform ECG monitoring by holding both sides of the device with both hands and transmit the ECG to a compatible smart phone or tablet PC. The device has been approved by the US Food and Drug Administration (FDA), Silver Spring, MD, USA [4]. Similar to the ACK detection mode, handheld devices include Omron Heartscan [5], Reka health [6], Zenicor ECG [7], etc. Smartwatch products are represented by Apple Inc.’s iWatch Cupertino, California. The detection algorithm adopted by it is the first algorithm approved by the FDA for the consumer market [8]. Users achieve single-lead ECG detection through the detector on the back of the watch face and the digital crown on the side of the watch face with both hands. Similarly, Randazzo et al. [9] designed an ECG watch that can record ECG results and share them via email. The above ECG watch devices are susceptible to noise artifact interference of arm movement [10] and need to complete the detection through the two-hand lap joint with a long distance between the measured points, which will affect the normal activities of users.

Advances have been made in both scientific research and products for “chest lead ECG recording devices”. Nemati et al. [11] designed a double-lead-based ECG monitoring system, which is composed of three electrodes for signal acquisition. Two electrodes are attached to the chest and a reference electrode is attached to the waist. Yang, Z. [12] and Wang, Y. [13] both adopt a three-lead ECG detection structure and place electrodes in the chest to form a triangular structure to capture ECG signals. The electrodes used in the above monitoring systems [11,12,13] need to be kept at a certain distance to ensure the accuracy of the test results. In terms of products, the single-lead system ZioPatch [14] and Cardiostat [15] and T-shirt-type wearable ECG clothing [16] should be highly integrated, but the difficulty comes from the fact that the electrode patches of the monitor still need to be spaced more than 5 cm apart.

If the distance between electrodes or detection points in the existing ECG detection equipment can be further reduced, or the area required for detection can be reduced, then it will provide good inspiration for improving the integration of the out-of-hospital ECG technologies. There is no product or work that can realize single-position ECG detection on the human body surface at present; the related works do not provide a description of such system. Therefore, a single-position ECG system based on charge induction is proposed to realize ECG detection on the surface of the human body with only one electrode with a diameter of less than 2 cm, characterized by its small size and easy wearability, which avoids the trouble of two-handed lap detection or products with a large detection area when using such products.

This paper is organized as follows. The first section is the simulation work, where an ECG waveform detected in a single location is simulated by analyzing the electro-physiological activities of the human heart on the human body surface with COMSOL Multiphysics 5.4 software. In the next section, the structure of the system, including the hardware circuit and the supporting software of the upper computer, is presented, while the excellent performance of the system is measured and introduced to prove that it can meet the requirements of weak ECG signal detection. Finally, the system is used for static and dynamic ECG monitoring. At the same time, the correlation calculation of ECG signals obtained by the two detection methods is carried out by comparing with the standard single-lead control test, which proves the reliability of our proposed system.

## 2. Simulation

The electroexcitation process of the heart affects all parts of the body and causes the potential difference of the body surface, which leads to the ECG [17]. In other words, for routine ECG detection, at least two or more detection points are needed to be placed on the body to form a single-lead or multi-lead ECG system. However, if there is only one detection point between the system and the body, what we will later refer to as single-position detection, traditional ECG detection cannot be carried out. Therefore, the ECG signal detected through only a single point on the body is different from the conventional ECG signal.

In order to test the feasibility of the single-point ECG detection method proposed in this paper, COMSOL Multiphysics 5.4 is used to simulate the electrophysiological activities of the human heart, observe the change curve of the bioelectrical signal on the body surface obtained from the single detection point, and make a qualitative analysis of the simulation results.

**Figure 1:**
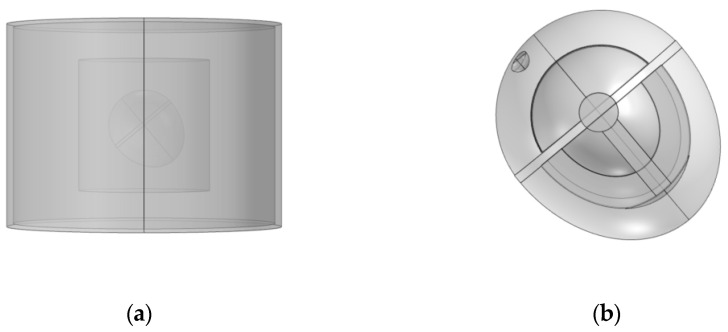
Three-dimensional heart model cross-section ((**a**) is the overall structure of the model, (**b**) is the details of the heart model).

Referring to a three-dimensional heart model structure proposed by Sovilj, S. et al. [18], the model is built as shown in the following figure. Figure 1a is the overall structure of the model, showing the electrode, trunk, lung, and heart from outside to inside. Figure 1b is a detailed diagram of the heart model. The proposed heart consists of eight parts, including the sinoatrial node, the atria, the atrioventricular node, the HIS bundle, the bundle branches, the Purkinje fibers, the ventricles, and the blood chambers.

In the model, partial differential equations are used as the physical field, and the FitHugh–Nagumo equation is used to simulate the conduction of cell action potential [19]. For each region of the heart, they are defined according to

(1) $$\frac{\partial u_{1}}{\partial t}=\Delta u+\left(\alpha -u_{1}\right)\left(u_{1}-1\right)u_{1}+\left(-u_{2}\right)\;\frac{\partial u_{2}}{\partial t}=\varepsilon \left(\beta u_{1}-\gamma u_{2}-\delta \right)$$

where $u_{1}$ is the action potential, $u_{2}$ is the gate variable, α represents the excitation threshold, ε denotes excitability, and β, γ, and δ are parameters that affect the resting state and dynamics of the system.

The blood chambers of heart, trunk, and lung in the model are defined as the passive volume conductor region; this means that the source term of the partial differential equation is 0, and the other part is the active volume conductor region where the partial differential equation term needs to be added. The parameters of each active region equation term are finally obtained through parameter adjustment, as shown in Table 1 below. The x of epsilon_x from 1 to 7 refers to the epsilon values of the sinoatrial node, the atria, the atrioventricular node, the HIS bundle, branch of bundles, the Purkinje fibers, and the ventricles, respectively, while the epsilon values of the remaining regions and parts are epsilon_0.

The diffusion coefficients in the partial differential equation are set according to the actual characteristics of each organ and structure of the human body [19]. Then the simulation results are adjusted to obtain the diffusion coefficient set in all parts of the model, as shown in Table 2 below.

**Figure 2:**
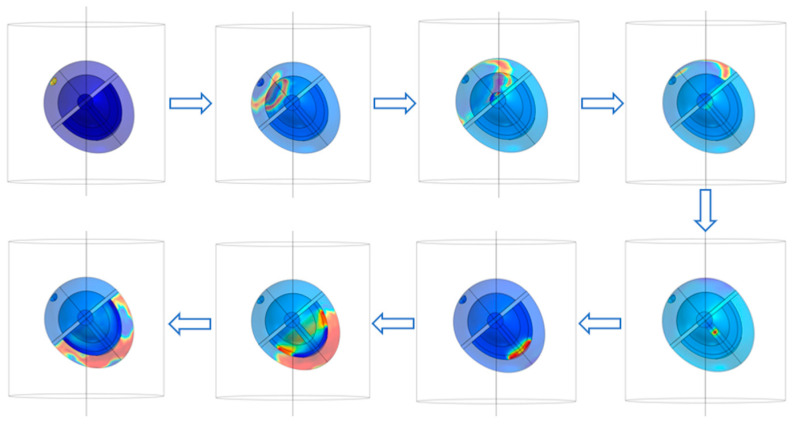
Action potential conduction in the heart.

The sinoatrial node in the heart is stimulated and boundary conditions and mesh are set for the above model to carry out the simulation of the complete ECG cycle. The conduction of action potential is shown in Figure 2. The electrophysiological characteristics of the heart are expressed as the excitatory function of the heart, which forms the generation and propagation of excitatory activity within the heart and leads to contraction. The warm color in Figure 2 represents the electric current generated by the electrical excitatory activity of cardiomyocytes. The electrical signal from the heart begins at the sinoatrial node, a crescent-shaped type of heart muscle cell located around the superior vena cava in the right atrium. The SA node is a self-excited cell that can generate 60 to 100 action potentials per minute, and this action potential travels through the atrial muscle. The nonconductive barrier of fibrous tissue is located at the boundary between the atria and ventricles, while action potentials cannot travel directly across this barrier. Therefore, the only conduction path from the atria to the ventricles is the atrioventricular node located at the atria–ventricle boundary. After the action potential propagates to the atrioventricular node, it propagates through the HIS bundle, bundle branch, and Purkinje fibers in turn.

**Figure 3:**
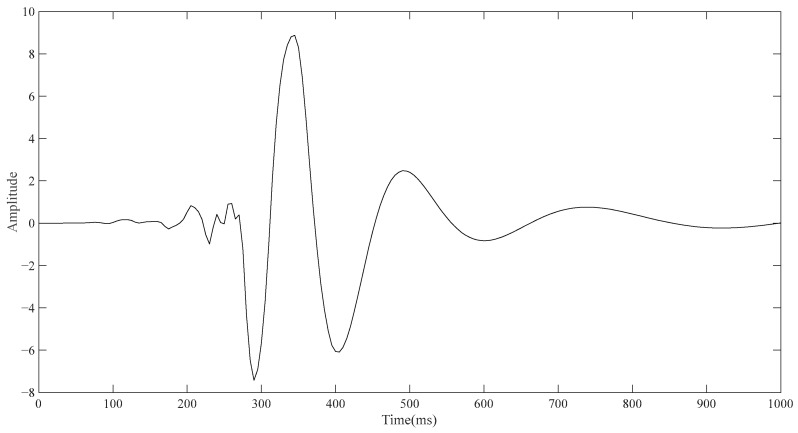
Simulation diagram of conventional ECG detection.

Since the electrode layer wrapped outside the torso will affect the simulation result, the outermost electrode layer is not added in the conventional ECG detection simulation, so the model consists of the torso, lung, and heart from the outside to the inside in turn. The lower left vertex of the torso in Figure 1a is taken as the reference point, and the upper left vertex of the torso is taken as the detection point. The point diagram is drawn using the built-in function of COMSOL, and the result is derived and then drawn using MATLAB. As shown in Figure 3, the approximate change curve of the body surface ECG signal can be obtained through simulation, including PQRST characteristic bands, although accurate results cannot be achieved in terms of signal amplitude and time.

**Figure 4:**
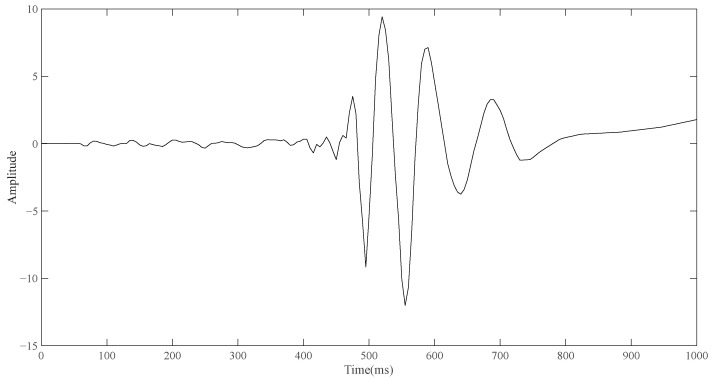
Simulation diagram of single position detection.

After the simulation of routine ECG detection is completed, the model containing the electrode layer is used for simulation. Similarly, the upper left vertex of the front side of the electrode layer in the model (the point contacting with the torso) is taken as the detection point. In the case that no reference point is added, the point diagram is also drawn through the built-in function of COMSOL, and the result is derived and drawn in the same way. As shown in Figure 4, the change curve of bioelectrical signals on the surface of the body obtained by single-pulse detection is a curve similar in shape to a wave.

Although it is not possible to quantitatively analyze the single-position ECG signal and conventional ECG signal in terms of amplitude and time through the simulation results, what can be seen is that the shape of the two curves is different, and the shape of the signal detected by the single-position ECG system is more similar to the wave. If the ECG detection can only be carried out by placing a single point of a small electrode device on the body surface, the optimization direction of the portable ECG detection equipment can have good detection capability. Based on the simulation results and the investigation of the ECG technologies, an ECG detection system is proposed which can be performed at a single position on the human body surface.

## 3. Systematic Design

The single-position detection system consists of three parts: the electrode with the charge sensor, the ECG acquisition circuit board, and the upper computer. The working process of the system is described as follows.

The charge sensor on the opposite side of the electrode converts the target charge induced by the detecting surface of the electrode into a voltage signal. The ECG acquisition circuit board then converts the voltage signal to digital and transmits it to the upper computer. The upper computer analyzes, saves, and processes the signal, displays the processed signal, and finally obtains the ECG waveform. The most significant advantage of this system is that it can convert the human charge induced by the detection surface of the electrode into voltage signals through the charge sensor, so it can finish the ECG signal detection at a single position of the human body and is not limited to two or more positions. The following sections describe the three parts of the system and show their performance.

### 3.1. Front-End Sensing Module

**Figure 5:**
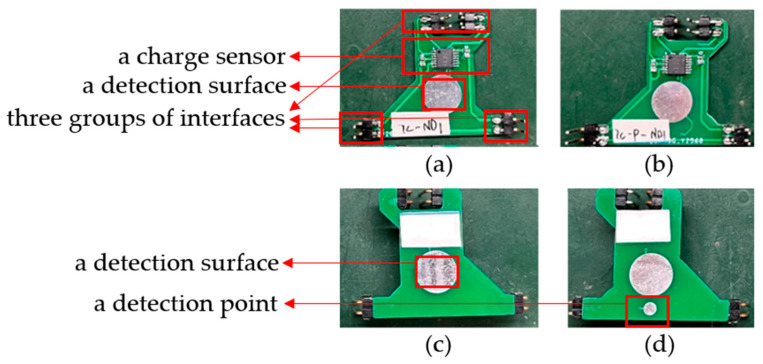
Front-end sensing module ((**a**,**b**) are the back side of one electrode and modified electrode, respectively; (**c**,**d**) are the front side of one electrode and modified electrode, respectively).

The electrode structure is later referred to as the front-end sensing module. As shown with one electrode in Figure 5a,c below, the front side (the side in contact with the human body) includes a detection surface, and the back side includes a charge sensor [20], a detection surface, and three groups of interfaces. The input capacitance and impedance of the charge sensor are about 10 pF and 150 GΩ, respectively, and the short-circuit noise is about 0.9 μV, so it can effectively sense the surface charge signal of the human body. The detection surface is used to sense the human charge at the fixed position, and the charge sensor is used to convert the induced human charge into voltage signals. The three groups of pin interfaces are used to transmit the signal, supply power to the charge sensor, and connect the electrode with the reference ground of the circuit board. In addition, an improved electrode form is also developed to suppress the interference caused by the normal activities of the measured subjects. As shown with one modified electrode in Figure 5b,d below, a referenced detection point is added next to the detection surface on the front of the electrode, and the signals obtained from them are differentiated, which can also achieve the target of detection. It should be pointed out that the size of the electrode is about 31 mm × 33 mm, in which the diameter of the circular detection surface is 10 mm, the diameter of the detection point is 3 mm, and the whole detection area can be compressed within the circular area with a diameter of less than 20 mm.

### 3.2. Back-End Acquisition System

**Figure 6:**
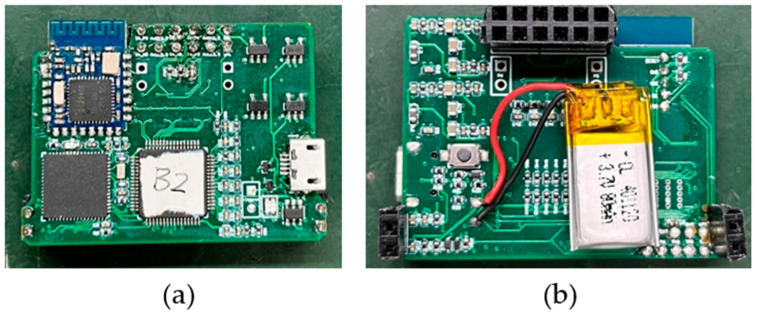
Back-end acquisition system ((**a**) is the front of the circuit board; (**b**) is the back of the circuit board).

The circuit board of the ECG acquisition system is called the back-end acquisition system. As shown in Figure 6, it includes the ADC module, MCU module, Bluetooth module, and power management module. The ADC module uses TI company’s 24-bit high-resolution integrated ADS1298 chip. Multi-channel ECG signals are input to the ADS1298 internal ADC in differential form for conversion and a novel front-end design is used [21]. The microprocessor chip MSP430F5528 is used to process and transmit the sampled data of ADS1298, and the low-power Bluetooth CC2541 is used to communicate with the upper computer to realize real-time wireless transmission of the collected data. The size of the circuit board is 41 mm × 32 mm. The system plate is connected by needle arrangement with the charged sensor electrode. After assembly, the system is light and small, and it is convenient to be placed on the body for ECG detection.

After the combination of the electrode and back-end acquisition system, the total size of the system is about 41 mm × 32 mm × 15 mm, and the weight of the system is about 12 g.

### 3.3. Upper Computer

**Figure 7:**
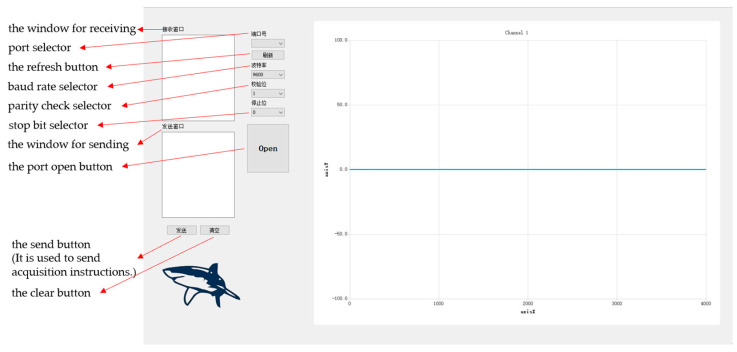
The upper computer.

As shown in Figure 7, the upper computer, which is developed independently by our laboratory and based on Qt software, is suitable for a variety of ECG test application scenarios. It can realize the functions of data receiving, processing, and real-time display and can store the received data in text format, which is convenient for us to filter and reduce the noise of the detected data.

### 3.4. Electrical Characteristics

The performance test of our system mainly includes a frequency response test, short circuit noise test, system common mode rejection ratio test, and system working power test. In order to improve the accuracy of these tests, 10 systems are used for testing and we average their results.

**Figure 8:**
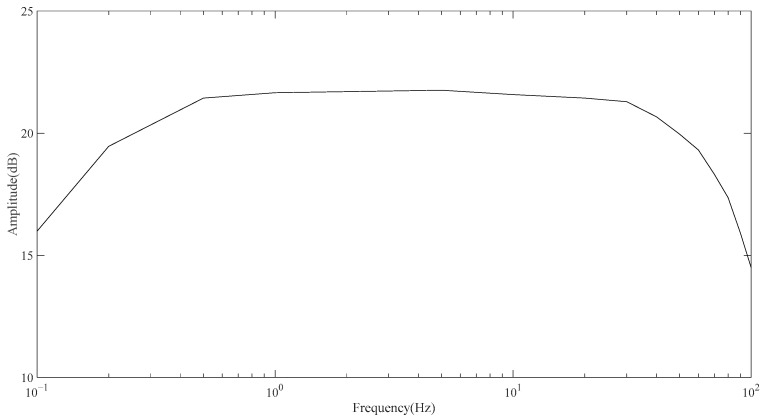
System frequency response curve.

For the frequency response test, the test signal is inputted from the detection surface of the front-end sensing module, and the output signal is saved by the upper computer. The test adopts the tracing method. It should be noted that the test signal is the sine wave signal with a peak-to-peak value of 100 mV generated by the signal source. Figure 8 shows the final measured system frequency response. It can be seen from its frequency response that the IF gain of the system is 21.76 dB and the bandwidth ranges from 0.18 Hz to 67 Hz. Generally, the frequency of ECG signals is distributed in the range from 0.01 Hz to 100 Hz, and 90% of the energy of ECG signals is concentrated in the range from 0.25 Hz to 35 Hz [22], so the performance of this system can meet the requirement.

For the input short-circuit noise test, the signal is inputted from the front-end high-resistance chip, and the input end is short-circuited. The equivalent input short-circuit noise is 1.74 μV on average, which meets the requirements of weak ECG signal detection.

For the common mode rejection ratio test, the signal is inputted from the input end of the back-end acquisition system, and output signal is saved by the upper computer for post-processing. Common mode rejection ratio measurements are taken under the conditions of a common mode input 50 Hz sinusoidal signal (amplitude 1000 mV) and differential input 50 Hz sinusoidal signal (amplitude 100 mV), and the average common mode rejection ratio is higher than 90 dB.

To test the working power of the system, a 3.6 V regulated power supply is used to supply power to the whole circuit system. The working power of the circuit system is tested before and after data acquisition and transmission is started. However, the difference of the system power before and after transmission is not obvious, and the average power is about 82.8 mW.

## 4. Tests and Analysis

In order to test the reliability of the single-position ECG detection system, the system is used to finish static and dynamic data collection. These results are processed and analyzed.

Static data collection refers to the 5 min control test by using the single-position ECG detection system and the conventional ECG system to collect the volunteer’s ECG signal at the same time for the experimental group and control group, respectively, while dynamic data collection refers to the 8 min ECG detection of the volunteer by using the single-position ECG detection system, which includes the collection of 4 different gestures. In each group of static tests, the volunteer needs to remain seated for 5 min, while in each group of dynamic tests, subjects need to maintain a sitting position for 2 min, then move their arms to tap the keyboard or use the mouse for 2 min to simulate doing office work, then rotate their upper body left and right for 2 min by turning around in the seat, and finally step in situ for 2 min to simulate the walking state.

A correlation analysis of the static control test is carried out after collection, and the signal-to-noise ratio is calculated and analyzed on the data of different motion postures.

**Figure 9:**
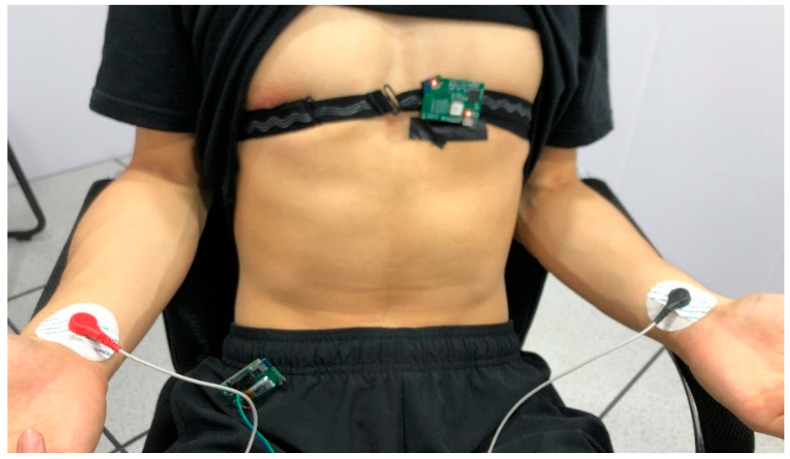
Test mode description.

As in Figure 9, for the experimental group, the system is anchored to the area around the unipolar limb lead V2 or V3 with the customized anti-slip band for men, and the system is fixed on the flat surface below the clavicle of the subject for women, because strong ECG signals can be obtained at these places [23]. At the same time, the medical gel is fixed on the wrists of both hands, and the conventional single-lead ECG signal of the control group is obtained by connecting the lead wire to the conventional ECG system. For dynamic data acquisition, the control group fixed on the wrist can be removed.

### 4.1. Heart Rate Correlation Characterization Method

A heart rate graph is a representation of the instantaneous heart rate of an individual in a period of time. The interval from one heartbeat to the next is converted into a value of heart rate per minute, which can represent the change trend of an individual’s heart rate. It is assumed that the distance between adjacent peaks is n, and the time interval of signal sampling is T. The heartbeat cycle is n×T, and the heart rate FHR = 60/n×T (bpm). The instantaneous heart rate calculated from each interval can be drawn into a curve with time as the horizontal coordinate and heart rate as the vertical coordinate, which can be called the heart rate curve, namely the heart rate graph.

Combined with the concept of the heart rate graph, the ECG acquisition method proposed in this paper and the detection method of the standard single-lead method are used to collect the ECG signals of the same subject. We record the two channels of ECG data, extract the peak value of QRS, and draw the two detection methods to obtain the heart rate graph and conduct correlation analysis with the standard single-lead signal as the control group. In this way, the reliability and data accuracy of the single-position ECG detection method can be evaluated.

### 4.2. Data Analysis

For the first set of tests, our ECG detection system with one electrode and a conventional ECG detection system are simultaneously used to collect the volunteer’s signal in a static state for 5 min for the experimental group and control group, respectively. It can be seen from experiments that the signal collected by the single-position ECG detection system with one electrode is more susceptible to baseline drift interference. However, considering that 90% of the energy in the ECG signal ranges from 0.25 Hz to 35 Hz, and in order not to filter the effective signal as much as possible, the high-pass filtering method with too high a cutoff frequency cannot be adopted in this test group. The filtering methods adopted here are 0.5 Hz high-pass, 50 Hz notch, 100 Hz notch, and 100 Hz low-pass.

**Figure 10:**
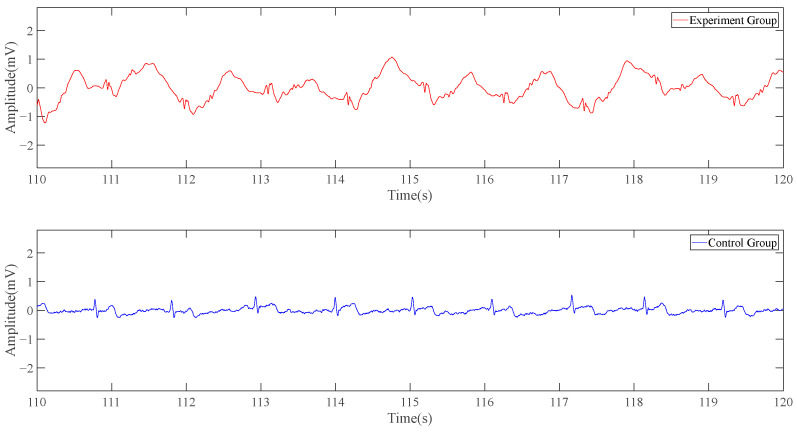
Result of static data (experimental group’s signal is collected by the system with one electrode).

The following is a 10-s signal segment of the data. In Figure 10, the red line represents the experimental group, namely the electrocardiogram collected by the single-position ECG detection system’s result, while the blue line represents the control group, namely the conventional ECG system’s result. The experimental group’s curve is similar to the result obtained in the simulation part, with the wavy part from the more obvious baseline drift. The number of spikes produced by the potential changes in the red line and the blue line within 10 s is the same, and there is an obvious high correlation in the time domain. Although the red line does not have the same ECG characteristic band as the blue line, it is periodic and has obvious peaks and troughs.

**Figure 11:**
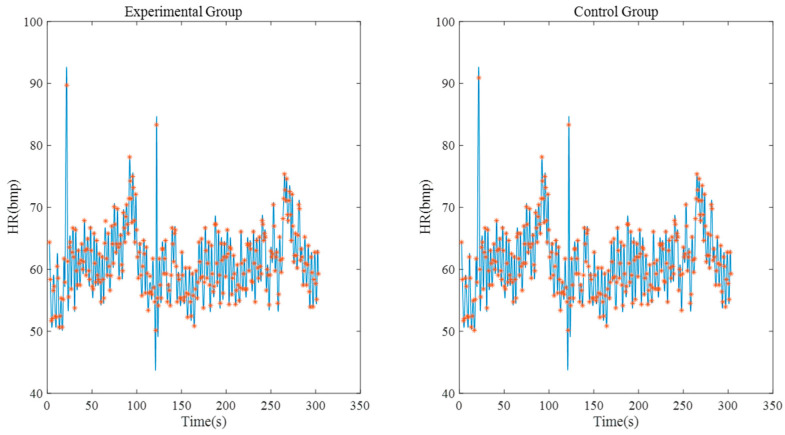
Heart rate correlation analysis (experimental group’s signal is collected by system with one electrode).

Figure 11 shows the heart rate correlation between the experimental group and the control group within 5 min. After calculation, it can be seen that the correlation coefficient between the experimental group and the control group reaches 0.9698 and the deviation of the maximum/minimum heart rate is within 1 bpm.

Similarly, a set of control tests is carried out by using the system with one modified electrode. As our system with one modified electrode uses one reference point to remove the baseline and interferences, the characteristics of the signal detected is basically consistent with those detected by conventional ECG, so this test does not need to strengthen the filtering of baseline drift. In order to retain the ability of most ECG signals, the cutoff frequency of high-pass filtering is adjusted to 0.25 Hz.

**Figure 12:**
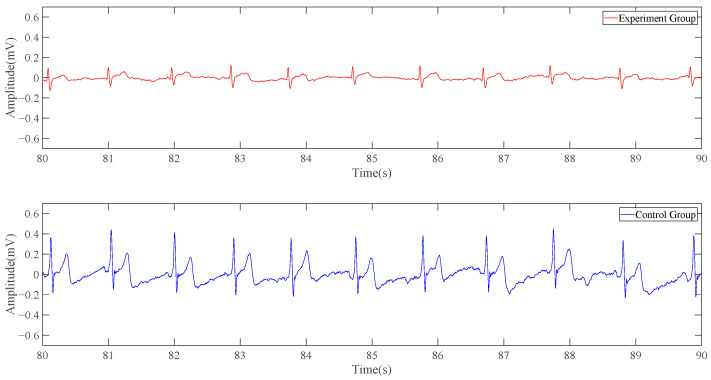
Result of static data (experimental group’s signal is collected by system with one modified electrode).

As can be seen from Figure 12, the one-position ECG detection system can obtain a complete and clear signal of the QRST band as the conventional ECG detection system does, but in terms of amplitude, the red line is significantly smaller than the blue line.

**Figure 13:**
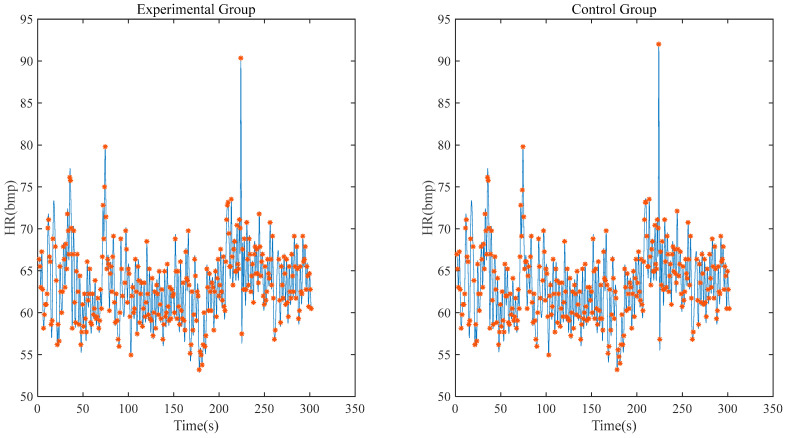
Heart rate correlation analysis (experimental group’s signal is collected by system with one modified electrode).

The following Figure 13 shows a 10-s signal and heart rate correlation, with a calculated correlation coefficient of 0.9802.

**Figure 14:**
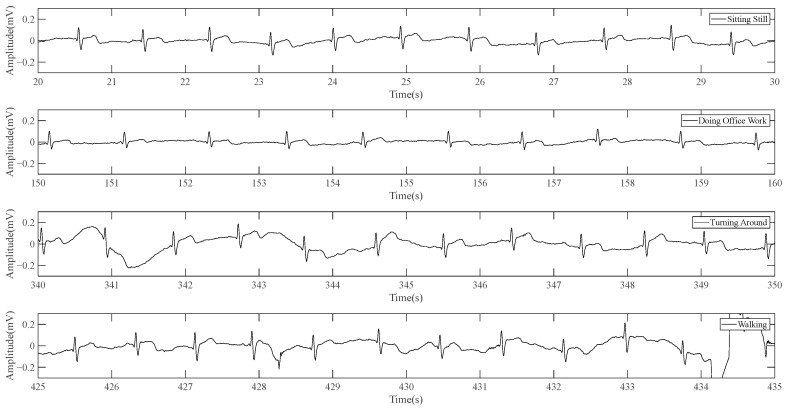
Result of dynamic data (signal is collected by system with one modified electrode).

Then the system with one modified electrode is used to conduct a set of dynamic data acquisition attempts to test the system’s anti-motion interference ability. Figure 14 shows 10 s of steady signal when the volunteer is in different postures: sitting, doing office work, turning around, and walking in situ. The filtering methods adopted in this test are 0.25 Hz high-pass, 50 Hz notch, 100 Hz notch, and 100 Hz low-pass.

Our system is used to consistently detect the ECG signal for about 8 min; sometimes the baseline of the signal fluctuates due to poor contact between the device and the body, caused by motion. Compared with the results of static ECG monitoring with few unrecognizable signals, there are about 20% unrecognizable signals when the subject performs the dynamic ECG monitoring experiment.

The results of all these experiments are from a male volunteer, 25 years old, who is in good health. 

To test the stability of the system’s detection, 20 volunteers, including 13 men and 7 women aged from 20 to 70, are also studied using a single-position ECG detection system with the modified electrode for static and dynamic ECG monitoring experiments.

For the static ECG monitoring experiment, the correlation coefficients between the signals collected by the single-position ECG detection system and the standard lead I signal are all above 0.9 and the mean deviation between the maximum and minimum heart rates is less than 1 bpm under the conditions of low environmental interference. For the dynamic ECG test, all data from the volunteers are calculated with a signal-to-noise ratio of more than 20 dB while in a sitting position. For the other three motion states, under the condition that the signal can be stably recognized, the calculated signal-to-noise ratio is greater than 12 dB. As for the difference between different genders, the most obvious manifestation in the results is that the maximum heart rate of women is almost higher than that of men, and there is no significant difference in the amplitude of the signal.

## 5. Discussion

The existing wearable ECG detection equipment is analyzed. A smartwatch requires lap joint detection with two hands, which affects normal activities, while ECG clothing uses a long distance between each electrode. If the distance between electrodes or detection points can be further reduced, or the area required for detection can be reduced, it can improve the integration of the out-of-hospital ECG equipment and can be more convenient for users to use.

The aim of the paper is to study and verify the feasibility of single-position ECG detection with only one electrode with a diameter of less than 2 cm. Firstly, COMSOL Multiphysics 5.4 software is used to simulate the electrophysiological activities of the human heart and obtain the simulation results that can be qualitatively analyzed, verifying the feasibility of single-position electrical detection. Then, the ECG detection system based on charge induction is introduced in detail, including the charge sensor, ECG acquisition circuit board, and upper computer, and the performance of the system can meet the requirements of weak ECG signal detection. In the last part, the system is used to perform static and dynamic ECG detection on the volunteer and perform heart rate correlation calculation to prove the reliability and data accuracy of the system.

## 6. Limitations

The single-position ECG detection system proposed in this paper is only a prototype system, and there are still some shortcomings and improvements. First of all, the work completed using COMSOL Multiphysics can only simulate sinus heart rate and cannot simulate various arrhythmias, such as PVCs and eventually atrial fibrillation. Secondly, the system cannot consistently detect the P-band, so the function to activate monitoring at the onset of disease is not developed. Thirdly, the heart rate of volunteers fluctuates normally all the time and the reliability of the system is not tested under the condition of a continuously high heart rate. Fourthly, the system is not tested during real activity, as well as the efficacy with different body morphologies. Moreover, adequate correlations during bradycardia and tachycardia should also be studied further.

## 7. Conclusions

In conclusion, the single-position ECG detection system proposed in this paper is different from the existing equipment. It achieves ECG detection in a circular area with diameter less than 2 cm and the results are accurate. The system is easy to wear and suitable for daily ECG detection.

On this basis, we can use multiple single-position ECG systems to form a multi-lead ECG system, which can obviously reduce the motion interference for the digital signal transmission with the single ECG systems instead of the analog signal with the electrodes. This will be studied in the future. In addition, in order to further increase the convenience of the ECG monitoring system and reduce the impact on users’ daily activities, it is of great significance to complete ECG detection on a single wrist of the subject to eliminate the lap joint detection with two hands, as required with current ECG watches, which is also a direction of future exploration.

## Figures and Tables

**Table 1:** Parameter values involved in partial differential equation terms.

Parameter	α	β	γ	δ	Epsilon_0	Epsilon_1
value	1	0.5	1	0	0	0.003
parameter	epsilon_2	epsilon_3	epsilon_4	epsilon_5	epsilon_6	epsilon_7
value	0.015	0.015	0.01	0.01	0.005	0.005

**Table 2:** Diffusion coefficients of partial differential equation terms.

Region	Body	Lung	Sinoatrial Node	Atrium	Atrioventricular Node
Value	0.2	0.2	0.1	0.15	0.1
Region	HIS bundle	Bundle branches	Purkinje fibers	Ventricle	Blood chamber
Value	0.1	0.1	0.3	0.1	0.7

## Data Availability

Not available.
